# Editorial

**Published:** 1851-01

**Authors:** 


### ATMOSPHERIC PRESSURE PLATES.

We have been repeatedly written to by members of the profession, in relation to sets and partial sets of teeth inserted on this principle. In the last number of the Register we gave two or three articles on this subject. First, an article by Dr. Flagg, giving a description of the lateral cavity plates. The plan proposed by Dr. Flagg, we have in two or three instances tried since the publication of his article, and one very similar several years before, but not with that success we fondly anticipated. The plan which we had tried previously, was an air chamber on either side within the alveolar border, and about half an inch from the ends of the plate. Chambers thus situated we still think would be more serviceable than any other. Keeping the plate in position on one side while masticating on the other; yet with our experience we have not found them any more useful, if as much so, as the central chamber recommended and patented by Mr. Gilbert—a description of which will be found in the last number of the Register, page 33, embraced in a report made by the Pennsylvania Association of Dental Surgeons.

The central cavity plate can be made available for the retention of temporary sets of teeth on the upper jaw; yet we do not find that the "rocking motion" alluded to by the report above named, is by any means entirely prevented; for when the alveolar ridge has receded by absorption an amount of pressure in chewing on either side which is greater than the suction, will of course rock the plate and let in the air. The air chamber here becomes a "pivot" on which at every occlusion of the teeth a lever force is applied, and the farther from this center the force is applied, the less weight will be necessary to overcome its adhesion. As the absorption therefore advances, the teeth will be less and less useful for mastication; and yet the teeth may be held in place and answer for all other purposes until absorption is completed. We have inserted teeth in this way, which held so firmly when the air was exhausted, that the draw on the palate was painful; and yet they were not really useful in mastication. We may therefore have an atmospheric pressure plate, and yet not have a serviceable set of teeth. We here repeat what we have before said in a former number of the Register, that the best atmospheric plates we have ever inserted, or the best and most serviceable we have ever seen, were plain plates. A plain plate which answers well the desired purpose, is better than any chamber plate. It may then be asked, why ever use the chambers? We answer, because we cannot always get the suction desired, and by the chamber we expect to secure the better retention of such plates.

The plan proposed by Dr. Palmer, and which may be found in the last number of the Register, page 35, we have not fully tested. When admissable these air chambers should, we think, be placed wherever depressions exist in the alveolar ridge.

In the present number of the Register we give another article on atmospheric pressure by Dr. James Fleming, Harrisburg, Pa.

We agree with the Dr. in many particulars, yet do not believe that wax is as certain to give a true impression as plaster. A very singular manner of removing the pressure of the atmosphere, so that the impression can be taken without injury from the mouth, was some two years since given by Dr. Dwinelle, in the American Journal, in a description of his method of taking plaster impressions. We had pursued the same plan in wax impressions, and gave description of the method in, we think, second volume Register. One, two, or three holes are made in the impression cup, and when the wax or plaster has been pressed to alveolar border and palate, an instrument is passed through these holes, passing up through the wax or plaster, till it touches the palate or gum, and lets in the air in the same manner as the tube. We prefer wax prepared by the heat of the spirit lamp or fire, to that softened in warm water, because we think it tougher and works better, and the sooner it receives the plaster the better. All swedging of the plate after it fits, we agree with the Dr., is injurious—one or two heavy blows will generally suffice. A thin plate may by constant use keep itself adapted to the changes of the mouth, but this generally destroys the articulation of the teeth.

In swedging the plate to the mouth, we have occasionally used over the cast representing the hard palate, a piece of lead foil. The gum on the alveolar ridge is often more yielding than the muscle which covers the palate, so that even the pressure of the wax may change the natural form of the parts, and this is one reason why plaster will sometimes give us a better impression than the wax. It is introduced when so soft as not by pressure to change the natural form of the parts to be covered by the plate.

The natural articulation of the teeth will always exert an influence on the service of atmospheric pressure sets of teeth. Teeth which project much, so that in biting pressure is made outside of the alveolar ridge, will not be so useful as when pressure is made more within. Dr. Ide, in the present number of the Register, has given some good directions on this subject.

---

### JONES, WHITE & CO.'S PREMIUM TEETH.

It affords us pleasure to say to our friends that these teeth are now for sale in Cincinnati. These gentlemen certainly deserve a great deal of credit for the improvements which they have made in their teeth. We have recently seen specimens of their gum, plate and pivot teeth which certainty come as near the natural organs as any we have ever seen. We have always been decidedly in favor of the enameled gum, thinking the color more durable than when painted. The inferior molar teeth, we are pleased to see, are made high on their palital edge, so as to meet square the superior teeth, without marring their beauty by the emery wheel. The fact is, our manufacturers are beginning to know that an inferior molar tooth is not to be used by the intelligent of the profession as a superior, or vice versa—that, indeed, in the artificial as well as natural, each tooth must take its appropriate place in the mouth and no other.

Dr. J. M. Brown, Dental Depot, corner Fourth and Walnut, is their agent for the sale of teeth. This gentleman, for his untiring industry in furnishing to the profession of the west every thing which is essential to their practice, deserves more than a passing act of gratitude. He has now by this new agency added to his assortment of teeth an additional amount, and we think that the profession will have no difficulty in getting all the different kinds of teeth they may require, from Stockton's justly celebrated teeth and Messrs. Jones, White & Co., both of which he keeps for sale.

While this arrangement will add very much to the accommodation of the profession, we do not see but it will be equally advantageous to the manufacturers. The object of a Dental Depot is to supply the profession with all articles needed in practice, and we are glad to see that Dr. Brown is determined to effect this object.

Diseases and Surgical Operations of the Mouth, by Jourdain. This excellent work we have just received from the enterprising publishers, Messrs. Lindsay & Blakiston, Philadelphia. The work is gotten up in their usual good style, good paper, and well bound, and should be in the hands of every dentist and physician.

The work is for sale by Derby & Co., Main street, Cincinnati.

---

### CONUNDRUM WHEELS.

We have been favored with a sample of these superior wheels by our friend Dr. E. Buckwell of Zanesville, Ohio. When kept wet they cut the teeth smooth and very rapidly, and in our estimation are far superior to the emery or stone. They are manufactured chiefly from the debris of the ruby and other precious stones. Dr. Buckwell is making arrangements for the importation of these valuable wheels from England. We know not what the price may be, but we would give three times as much for them as the emery.

---

Fire.—We are sorry to learn that the office of Drs. Holmes & Walters, of Lockport, N. Y., including furniture, stock of materials, instruments, several set of teeth nearly completed, library, clothing, &c., were all consumed by fire on 7th of last November. We understand that the loss principally falls on Dr. Holmes, who was not insured—loss from $1,000 to $1,200. His partner (Dr. Walters) was nearly covered by insurance.

---

Transylvania School of Dental Surgery.—We received last fall the circular of this Institution too late to be noticed in the October number of the Register. We sincerely wish success to every enterprise which shall advance the cause of dental science.

---

Errata from last number.—On page 19 6th line from the bottom read "hand" for "had."

On page 20 10th line from the bottom, read "entire" for "center.

